# Mitochondria‐Targeted ROS Scavenging Natural Enzyme Cascade Nanogels for Periodontitis Treatment via Hypoxia Alleviation and Immunomodulation

**DOI:** 10.1002/advs.202507481

**Published:** 2025-05-23

**Authors:** Yanfen Zheng, Liuzhou Mao, Qi Wang, Haohua Hu, Bahriman Xarpidin, Zheng Luo, Yun‐Long Wu

**Affiliations:** ^1^ Xiamen Key Laboratory of Stomatological Disease Diagnosis and Treatment Stomatological Hospital of Xiamen Medical College Xiamen 361008 China; ^2^ Engineering Research Center of Fujian University for Stomatological Biomaterials Xiamen 361023 China; ^3^ Fujian Provincial Key Laboratory of Innovative Drug Target Research and State Key Laboratory of Cellular Stress Biology, School of Pharmaceutical Sciences Xiamen University Xiamen 361102 China

**Keywords:** anti‐inflammatory, mitochondrial targeting, natural enzyme cascade, periodontitis, ROS scavenging

## Abstract

Periodontitis is a chronic inflammatory disease characterized by hypoxia, excessive oxidative stress, and immune dysregulation, leading to tissue destruction and bone resorption. Although antioxidants can reduce ROS and inflammation, most lack specificity and have a short residence time, limiting their effectiveness. Since mitochondria are the primary source of ROS, targeting mitochondrial ROS is a promising strategy for periodontitis treatment. However, this alone cannot address the complex “ROS‐inflammation‐hypoxia” cycle in periodontitis, requiring a more comprehensive approach. Here, a natural enzyme cascade nanogel (TPP‐SAT) composed of triphenylphosphine (TPP), superoxide dismutase (SOD), and catalase (CAT) via in‐situ polymerization is developed. TPP‐SAT targets mitochondrial ROS, converting ROS (such as H_2_O_2_ or ·O_2_^−^) into O_2_ through the enzyme cascade of SOD and CAT. This alleviates hypoxia, prevents oxidative damage, and restores the balance between pro‐inflammatory M1 and anti‐inflammatory M2 macrophages, reducing inflammation and immune dysfunction. TPP‐SAT breaks the “ROS‐inflammation‐hypoxia” cycle, inhibits alveolar bone resorption, and accelerates periodontal tissue regeneration. This approach offers a promising strategy for treating periodontitis and other chronic inflammatory diseases, with strong clinical potential.

## Graphical abstract

TPP‐SAT, a mitochondria‐targeted natural enzyme‐cascade nanogel, selectively scavenges mitochondrial ROS (e.g., H_2_O_2_/·O_2_^⁻^) and converts them into therapeutic O_2_ via enzymatic cascades, alleviating hypoxia and inflammation. It rebalances M1/M2 macrophage polarization, activates the Nrf2 pathway while suppressing NF‐κB, enhances antioxidant defenses, and promotes angiogenesis, thereby accelerating periodontal tissue regeneration.

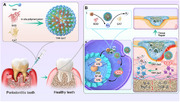

## 1 Introduction

Periodontitis is a chronic inflammatory disease caused by pathogenic bacteria, which is usually accompanied by hypoxia, oxidative stress, and immune response disorders.^[^ ^1^ ^]^ These complex pathological microenvironmental characteristics often lead to gingival redness and swelling, periodontal pocket formation, and even tooth loss in severe cases.^[^ ^2^ ^]^ Excessive oxidative stress and inflammatory response are the main factors leading to persistent periodontitis, which can cause a large amount of reactive oxygen species (ROS) generation and immune dysfunction, trigger vascular dysfunction and aggravate tissue hypoxia.^[^ ^3^ ^]^ Hypoxia will lead to mitochondrial dysfunction and increase the activity of the mitochondrial electron transport chain, leading to the production of superoxide (such as ·O_2_^−^) and other ROS, further aggravating the generation of ROS.^[^ ^4^ ^]^ Excessive ROS not only damages the mitochondria themselves, but also promotes the release of pro‐inflammatory factors by inducing macrophages to transform into pro‐inflammatory M1 macrophages,^[^ ^5^ ^]^ forming a vicious cycle of “ROS‐inflammation‐hypoxia”, and aggravating the level of inflammation in periodontal tissues. Therefore, it is crucial to eliminate excessive ROS, relieve hypoxia and anti‐inflammation to break the vicious cycle of the three to relieve periodontitis.

Mitochondria, as the energy metabolism center of cells, efficiently generate ATP through oxidative phosphorylation when oxygen is sufficient, but their electron transport chain is also the main source of ROS.^[^ ^4^, ^6^ ^]^ Under hypoxic conditions, the electron transfer of mitochondrial respiratory chain complexes (especially complex III) is blocked,^[^ ^4^, ^7^ ^]^ resulting in the accumulation of free radicals, which promotes the abnormal generation of ROS such as superoxide. This “hypoxic ROS burst” can aggravate oxidative stress through the HIF‐1α signaling pathway and form an inflammatory amplification effect.^[^ ^8^ ^]^ In recent years, antioxidants such as polyphenols, metal‐based nanozymes and natural enzymes have been widely used in the management of periodontitis to resist ROS and reduce inflammation.^[^ ^9^ ^]^ However, most ROS scavengers lack specific targeting and have a short retention time in the body, resulting in low ROS scavenging efficiency and unsatisfactory therapeutic effects. Currently, targeted removal of mitochondrial ROS has shown gratifying therapeutic effects in various inflammatory diseases,^[^ ^10^ ^]^ such as arthritis, enteritis and diabetes. Therefore, targeted removal of mitochondrial ROS may be the key to breaking the vicious cycle of “excessive ROS‐inflammation‐hypoxia”. Superoxide dismutase (SOD) and catalase (CAT) are one of the important antioxidant systems in cells. They can efficiently convert ROS such as O_2_^−^ and hydrogen peroxide (H_2_O_2_) into oxygen through enzyme cascade reactions,^[^ ^11^ ^]^ reducing ROS levels while alleviating hypoxia, achieving two birds with one stone. However, due to the complex structure, poor stability and large molecular weight of natural enzymes, how to stably deliver them to the mitochondria of periodontal cells to exert their effects is still a huge challenge.

**Scheme 1:**
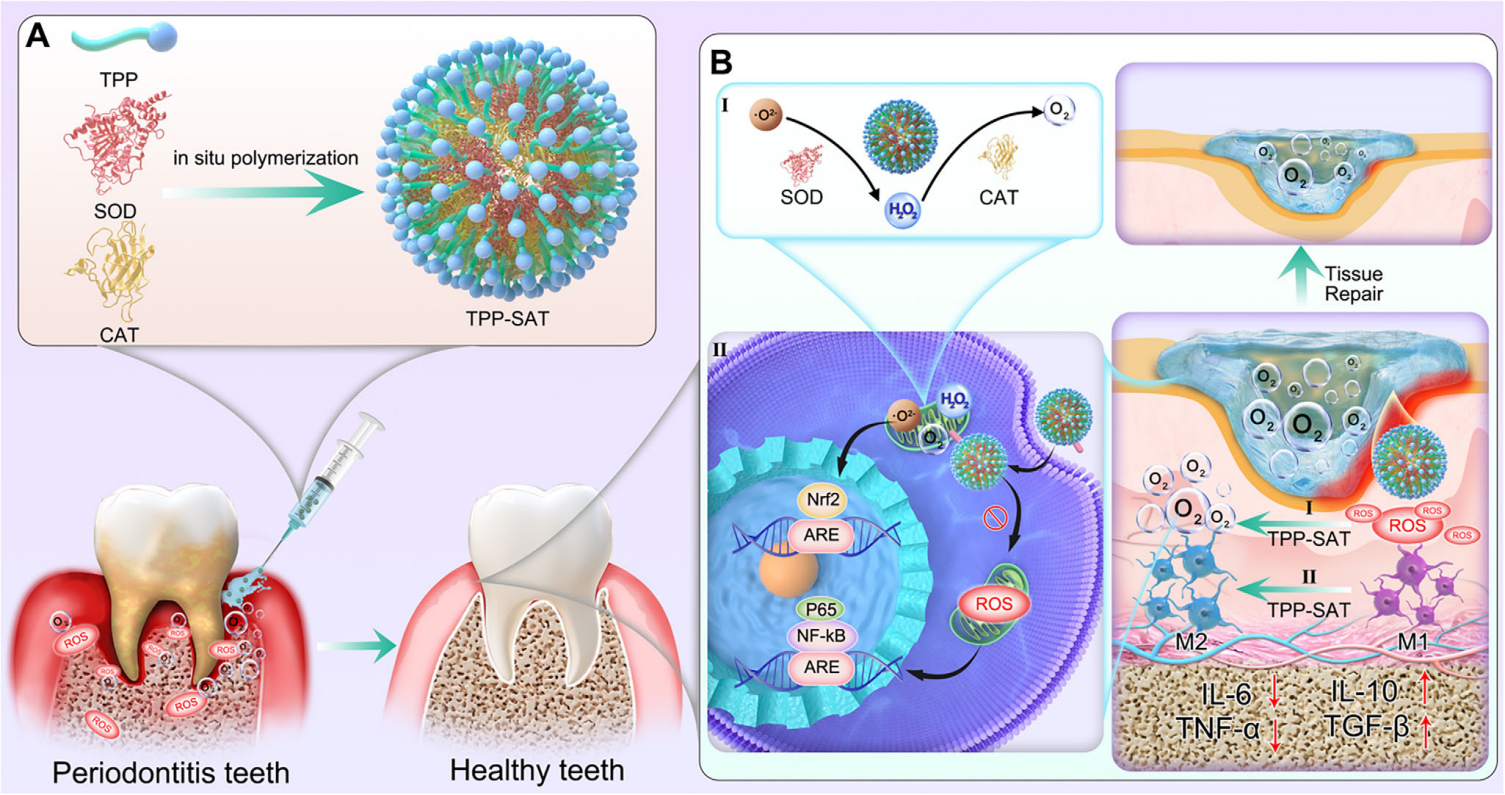
Synthesis and mechanism of action of TPP‐SAT. A) Synthesis scheme of TPP‐SAT. B) Mechanism of action of TPP‐SAT in cells.

In the study, we developed a natural enzyme nanogel (TPP‐SAT) that targets and removed mitochondrial ROS for innovative treatment of periodontitis based on the preparation method of natural enzyme preparations in the previous period of the research group,^[^ ^12^ ^]^ which is mainly composed of mitochondrial target head triphenylphosphine (TPP), SOD and CAT (**Scheme** **1**). The nanogel can effectively improve the stability of natural enzymes, and its nanosize effect can effectively shorten the spatial distance between SOD and CAT, and improve the enzyme cascade reaction efficiency between the two. Under the action of TPP, TPP‐SAT can accurately target mitochondria, and efficiently remove ·O_2_^−^ and H_2_O_2_ produced in the mitochondrial site and generate O_2_ through the cascade reaction of SOD and CAT, thereby alleviating the high oxidative stress and hypoxic environment in the periodontal cell. This will further help pro‐inflammatory M1 macrophages polarize to anti‐inflammatory M2 phenotype and reshape the immune environment, while the oxygen produced by the enzyme cascade reaction will help promote tissue repair and angiogenesis, restore the nutrient supply of periodontal tissues, and comprehensively improve the pathological microenvironment of periodontitis. More importantly, this natural enzyme preparation is simple to synthesize, easy to use, biosafe, and effective in treatment, which will provide new insights into the clinical treatment of periodontitis.

## 2 Results and Discussion

### 2.1 Fabrication and Characterization of TPP‐SAT Nanogel

**Figure 1:**
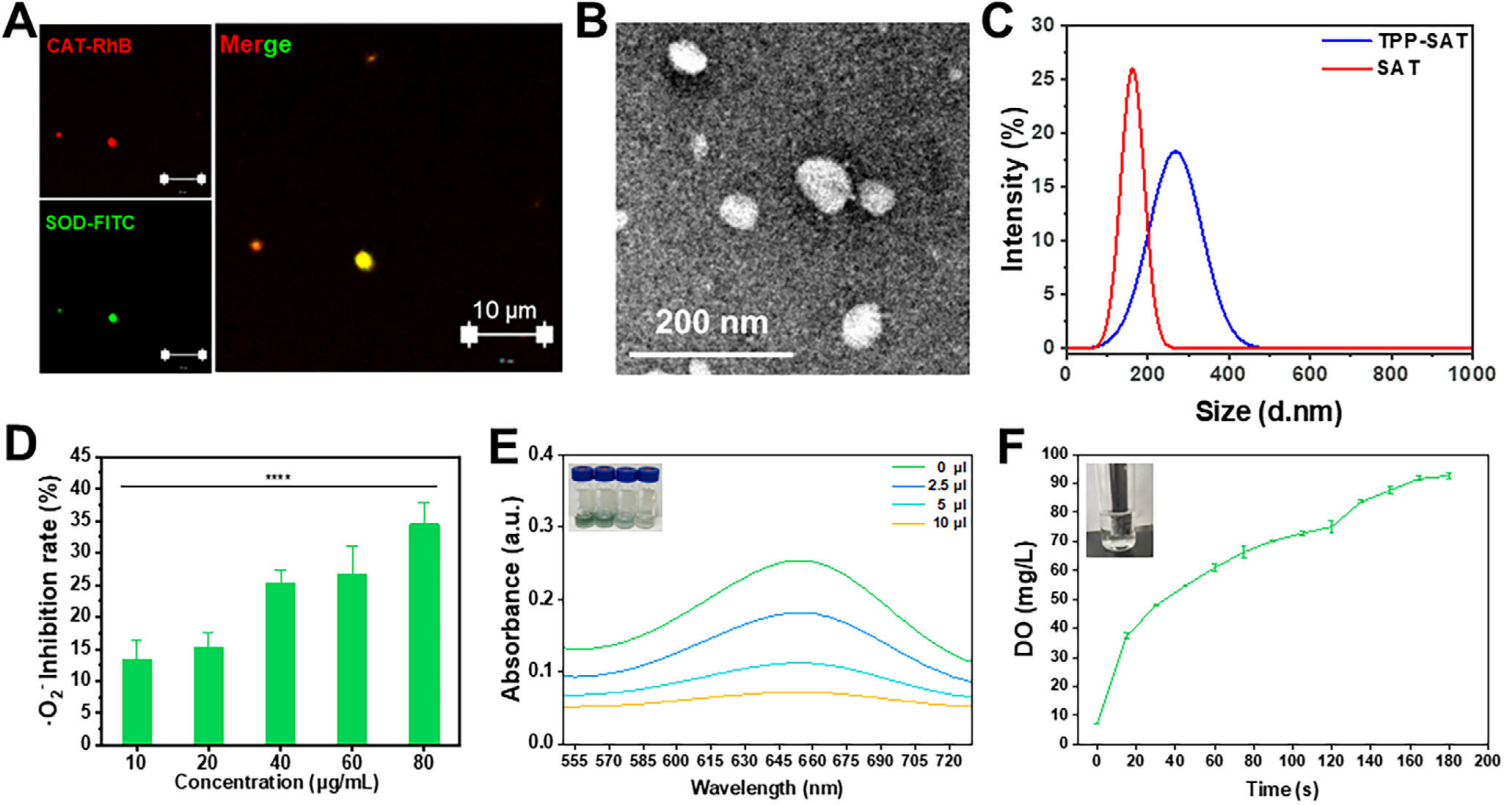
Fabrication and characterization of TPP‐SAT nanogel. A) The fluorescence imaging of TPP‐SAT (red signal represents CAT, green signal represents SOD) (Scale bar: 10 µm). B) TEM image of TPP‐SAT (Scale bar: 200 nm). C) Particle size distribution of SAT and TPP‐SAT. D) Measurement of superoxide dismutase (SOD) activity in TPP‐SAT. E) Assessment of H₂O₂ scavenging capacity of TPP‐SAT using TMB colorimetric assay. F) Quantification of O_2_ production from H₂O₂ decomposition by TPP‐SAT. Data are presented as the mean ± standard deviation (SD). Statistical significance was calculated by t‐test for comparison between two groups. ns: no significant, ****p < 0.0001.

TPP‐SAT was synthesized via free radical polymerization of TPP, SOD, and CAT. FTIR (Fourier Transform Infrared) analysis confirmed the occurrence of radical polymerization, as evidenced by the emergence of a distinct P‐C peak at 1112 cm⁻¹ in TPP‐SAT, along with the disappearance of characteristic double bond vibrations at 990 and 928 cm⁻¹ that were originally present in the TPP structure (Figure S1, Supporting Information). In addition, CLSM fluorescence imaging revealed significant colocalization of FITC (green fluorescence)‐labeled SOD and Rhodamine B (RhB, red fluorescence)‐labeled CAT, demonstrating successful conjugation of both SOD and CAT enzymes (**Figure** **1**A). TEM micrographs revealed that TPP‐SAT exhibited spherical morphology with an average diameter of ≈270 nm, demonstrating a noticeable size increase compared to SAT (Figure 1B,C). These experimental results collectively demonstrate the successful synthesis of TPP‐SAT.

Subsequently, the enzymatic activities of both SOD and CAT in TPP‐SAT were thoroughly validated. As shown in Figure 1D, quantitative analysis using a standard SOD activity assay demonstrated concentration‐dependent superoxide radical scavenging capability of TPP‐SAT. Furthermore, TMB‐based colorimetric assays revealed significant attenuation of the characteristic 652 nm absorption peak resulting from H_₂_O_₂_‐mediated TMB oxidation, confirming efficient H₂O₂ elimination (Figure 1E). Concurrently, dissolved oxygen measurements verified robust catalytic decomposition of H₂O₂ by the incorporated CAT enzyme, accompanied by substantial oxygen evolution (Figure 1F). These collective findings demonstrated excellent preservation of dual enzymatic activities in TPP‐SAT, establishing its suitability for subsequent biomedical applications.

### 2.2 Validation of the Biocompatibility and Mitochondrial Targeting Ability of TPP‐SAT In Vitro

**Figure 2:**
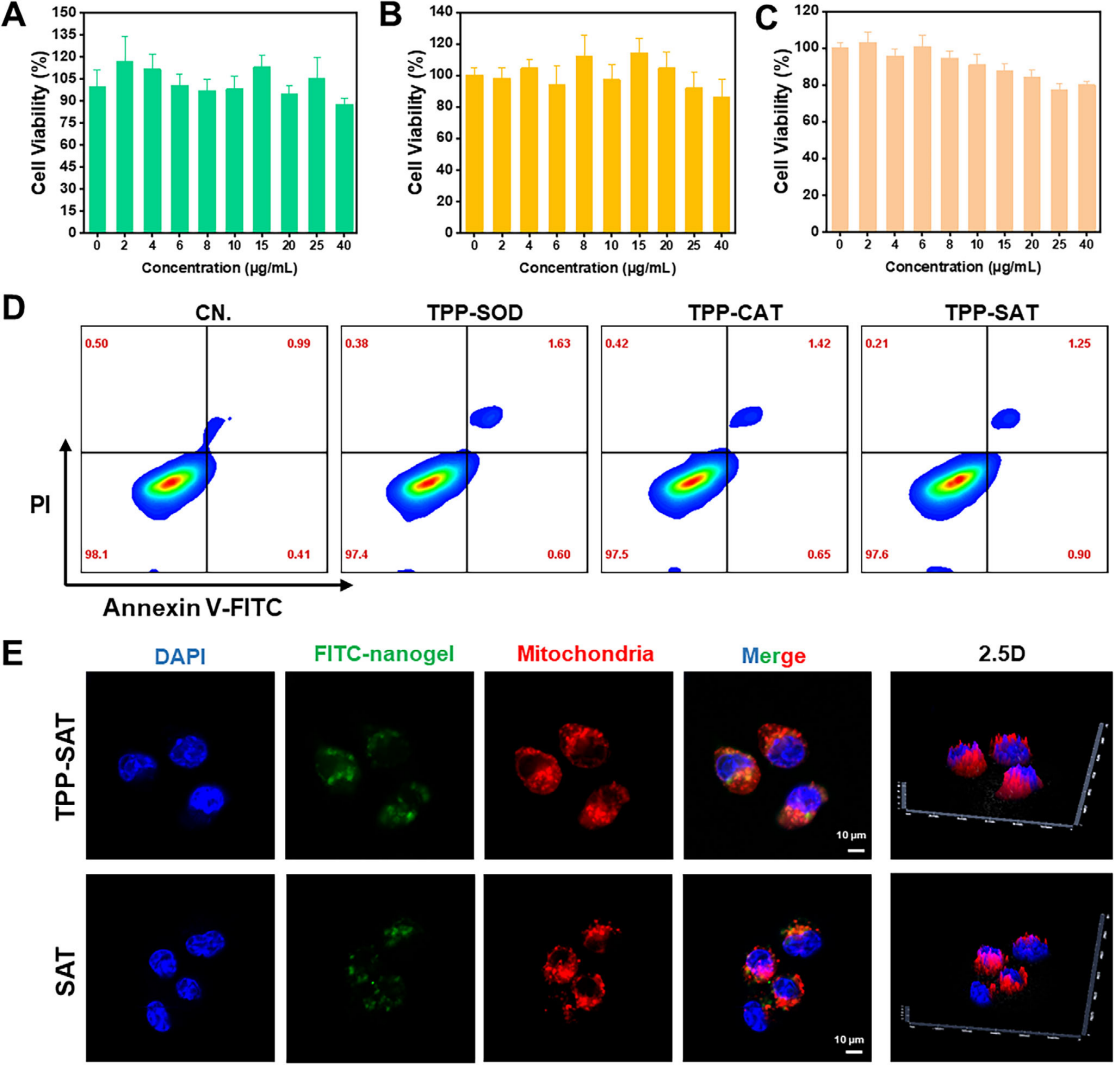
Biocompatibility and mitochondrial targeting ability study of TPP‐SAT. MTT assay to verify the cytotoxicity of TPP‐SAT in A) RAW 264.7 cells (*n* = 3) B) PDLSC cells (*n* = 3) and C) HUVEC cells (*n* = 3). D) Apoptotic cycle assay of RAW 264.7 cells after treatment with different groups of materials. E) Fluorescence experiments verified the mitochondrial targeting ability of TPP‐SAT and SAT (Scale bar: 10 µm).

The biocompatibility level is the most critical factor in assessing the potential of a material for translational applications. Therefore, the biocompatibility of TPP‐SAT was fully validated in this study by MTT experiments with three cell lines (RAW 264.7, PDLSC, and HUVEC). As shown in **Figure** **2**A–C, it is shown that the TPP‐SAT still did not show significant cytotoxicity even when the drug concentration of TPP‐SAT reached 40 µg mL^−1^. Moreover, the toxicity of TPP‐SAT was almost negligible at a administration concentration of 10 µg mL^−1^, so based on the studies at the material level and cytotoxicity level, the administration concentration of 10 µg mL^−1^ was selected for the next step of the experiments based on a comprehensive consideration. Subsequently, the cytotoxicity of the different groups of materials was investigated by means of a cell activity assay kit. And the cytotoxicity of each group of materials was almost negligible as observed by inverted fluorescence microscopy (Figure S2, Supporting Information). Similar conclusions to the above were also reached by apoptotic cycle assay of RAW 264.7 cells treated with different groups of materials and analyzed by flow cytometry (Figure 2D).

Subsequently, the mitochondrial targeting of TPP in the components of TPP‐SAT was verified by fluorescent staining. As shown in Figure 2E, it can be observed that FITC‐labeled TPP‐SAT is highly overlapped with mitochondria, while FITC‐labeled SAT did not show sufficient mitochondrial targeting ability, which provides precise scavenging of free radicals by TPP‐SAT nanogel. In conclusion, the above results demonstrated that TPP‐SAT nanogels have excellent biocompatibility and potential for periodontitis treatment, which provides strong conditions for their clinical application.

### 2.3 Validation of the Enzymatic Cascade Reaction and Related Active Functions of TPP‐SAT

**Figure 3:**
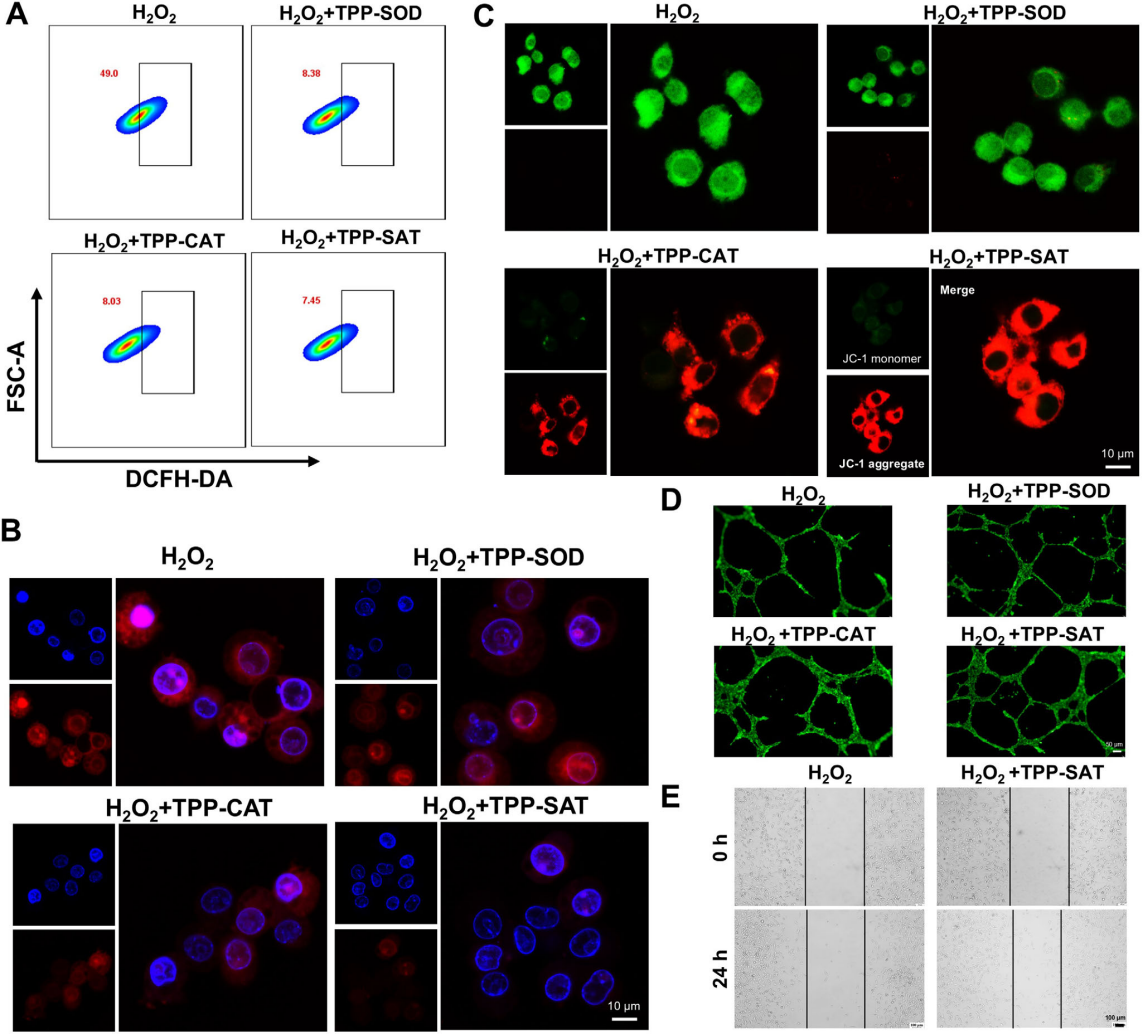
In vitro study of the action mechanism of TPP‐SAT nanogel. A) Flow cytometry validation of TPP‐SAT for free radical scavenging in vitro. B) The oxygen probe [Ru(dpp)_3_]Cl_2_ is used to detect the concentration of oxygen in the cell (a weaker red light indicates a higher concentration of oxygen) (Scale Bar: 10 µm). C) CLSM image of cellular mitochondrial membrane potential detected by JC‐1 fluorescent probe (Scale Bar: 10 µm). D) Angiogenesis image of HUVEC cells after treatment with different materials (Annexin‐V FITC staining) (Scale Bar: 50 µm). E) Validation of TPP‐SAT's ability to promote L929 cell migration (Scale bar: 100 µm).

After validating the biocompatibility and its mitochondrial targeting properties of TPP‐SAT, its enzyme cascade reaction related characterization will be validated in this section. It is well known that the natural enzyme SOD has the ability to catalyze the production of H_2_O_2_ from superoxide radicals, while CAT catalyzes the breakdown of H_2_O_2_ into O_2_. Therefore, the enzymatic cascade reaction of SOD and CAT can effectively scavenge free radicals and produce oxygen, which helps in inflammation repair. As shown in **Figure** **3**A, compared to the H_2_O_2_ group, the number of ROS‐positive cells in the H_2_O_2_+TPP‐SAT group was only 7.45%, which was one‐sixth of the value in the H_2_O_2_ group. In addition, there was also a decrease in the number of ROS‐positive cells in TPP‐SAT compared to the single treatment group, which proves that the enzyme cascade reaction has a better application advantage. And the conclusions obtained from ROS fluorescent staining appealed to consistency (Figure S3, Supporting Information). Subsequently, we validated the activity of the natural enzyme CAT in TPP‐SAT by means of an oxygen fluorescent probe. As shown in Figure 3B, the red fluorescence of the oxygen probe was not quenched in the H_2_O_2_ group and H_2_O_2_+TPP‐SOD because there was no significant oxygen production, whereas the red fluorescence of the oxygen probe in the H_2_O_2_+TPP‐CAT and H_2_O_2_+TPP‐SAT groups was quenched due to the production of large amounts of oxygen from CAT‐catalyzed H_2_O_2_. And high levels of ROS cause a decrease in cellular mitochondrial membrane potential (MMP) and lead to apoptosis. As shown in Figure 3C, staining by JC‐1 fluorescent probe revealed that the cells in the H_2_O_2_+TPP‐CAT and H_2_O_2_+TPP‐SAT groups showed obvious red fluorescence, demonstrating that their MMP did not show a significant decrease. In contrast, the cells of H_2_O_2_ group showed obvious green fluorescence, demonstrating that a significant decrease in their mitochondrial membrane potential occurred. Interestingly, the mitochondrial membrane potential of the cells in the H_2_O_2_+TPP‐SOD group also showed a decrease, which appears to be a discrepancy with the conclusions obtained by flow cytometry, which may be a decrease in the ROS‐positive cells due to the scavenging of superoxide radicals by the TPP‐SOD in the ROS assay, whereas in the JC‐1 assay it is a decrease in the mitochondrial membrane potential due to the mitochondrial oxidative stress of cells as a result of H_2_O_2_. And the JC‐1 fluorescence quantification experiments are also consistent with the conclusions reached on appeal (Figure S4, Supporting Information).

In addition, Nrf2 as well as P65 are the relevant proteins for anti‐oxidative stress as well as oxidative stress pathway in cells, respectively. The P65 as well as Nrf2 expression was observed by immunofluorescence staining assay, it can be found that the expression of the anti‐oxidative stress protein Nrf2 in the H_2_O_2_+TPP‐SAT group was significantly elevated, while the expression of the oxidative stress protein P65 was significantly decreased compared with the other groups (Figure S5, Supporting Information). This demonstrated that TPP‐SAT had a significant effect on removing cellular oxidative stress. And oxygen therapy has been shown to promote angiogenesis (the formation of new blood vessels). Therefore, the ability of TPP‐SAT to promote vascular regeneration was verified by the HUVEC tube‐forming assay. As shown in Figure 3D, the H_2_O_2_+TPP‐SAT group expressed a significant ability to promote angiogenesis, whereas the effect of the monotherapy group was not significantly enhanced. In addition, cell migration assays demonstrated that H₂O₂+TPP‐SAT treatment significantly enhanced L929 fibroblast proliferation compared to H₂O₂ treatment alone, suggesting its potential to accelerate wound healing processes (Figure 3E). The above experimental results demonstrate that TPP‐SAT has the ability to efficiently scavenge free radicals and generate large amounts of oxygen, thereby promoting neovascularization and contributing to the repair of periodontal tissues.

### 2.4 Validation of TPP‐SAT to Improve the Inflammatory Immune Microenvironment

**Figure 4:**
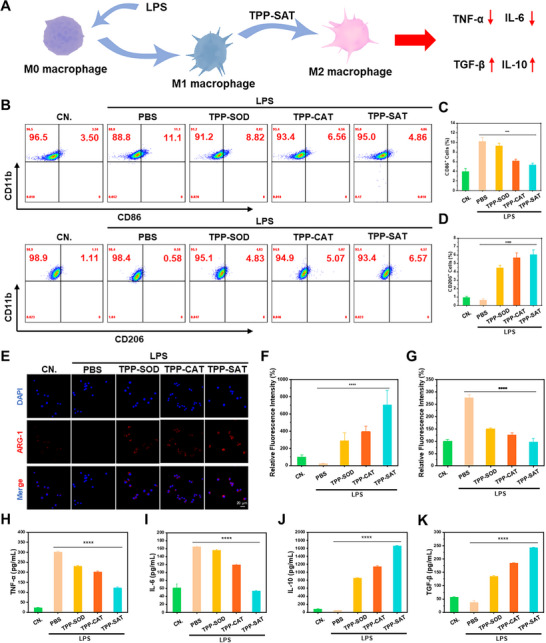
TPP‐SAT reverses the inflammatory microenvironment. A) Schematic illustration of an experiment in which TPP‐SAT reversed M1 pro‐inflammatory macrophages to M2 anti‐inflammatory macrophages. B) Flow cytometry analysis of CD86 and CD206 expression on the surface of macrophages. Quantitative analysis of surface expression levels of C) CD86 and (D) CD206 on macrophages under different treatment conditions. E) Immunofluorescence staining of ARG‐1 on the surface of macrophages under different treatment conditions (Scale Bar: 20 µm). Quantitative fluorescence analysis of F) ARG‐1 and G) iNOS surface expression on macrophages under different treatment conditions. Quantitative analysis of cytokine concentrations H) TNF‐α, I) IL‐6, J) IL‐10, and K) TGF‐β in macrophage culture supernatants under different treatment conditions. Data are presented as the mean ± standard deviation (SD). Statistical significance was calculated by t‐test for comparison between two groups. ns: no significant, **p < 0.01, ***p < 0.001, ****p < 0.0001.

Macrophages at the inflammatory site of periodontitis are polarized to the pro‐inflammatory type due to infection by pathogens such as bacteria, thus further aggravating inflammation. Therefore, it is hoped that TPP‐SAT can effectively reverse this inflammatory immune microenvironment by scavenging free radicals and turning pro‐inflammatory macrophages into anti‐inflammatory ones. The specific validation scheme is shown in **Figure** **4**A. The macrophage surface proteins CD11b (macrophage marker), CD86 (M1 macrophage marker) as well as CD206 (M2 macrophage marker) after treatment in different ways were stained and detected in flow cytometry. As shown in Figure 4B–D, TPP‐SAT not only reduces the proportion of M1‐type macrophages but also promotes their repolarization into M2‐type macrophages. Specifically, the percentage of M1‐type macrophages decreased from 11.1% to 4.86%, while the proportion of M2‐type macrophages increased from 0.58% to 6.57%. And the quantitative data are also consistent with the conclusions obtained by flow cytometry. Furthermore, immunofluorescence staining of ARG‐1 (M2 macrophage marker) and iNOS (M1 macrophage marker) in differently treated cells (Figure 4E; Figures6, Supporting Information) revealed that the TPP‐SAT administration group exhibited a significant increase in ARG‐1 expression and a marked reduction in iNOS levels, consistent with the experimental findings shown in Figure 4B   . Additionally, the results obtained from quantitative fluorescence analysis aligned with the immunofluorescence data, further validating the observed effects (Figure 4F,G). Subsequent analysis of inflammatory cytokines in the supernatant of different treatment groups revealed that TPP‐SAT administration significantly increased the concentrations of anti‐inflammatory cytokines (IL‐10 and TGF‐β) while markedly reducing pro‐inflammatory cytokines (IL‐6 and TNF‐α) (Figure 4H–K). These results robustly demonstrate TPP‐SAT's ability to modulate the inflammatory microenvironment, suggesting that its therapeutic effects on periodontitis may be mediated through amelioration of periodontal inflammatory conditions.

### 2.5 Validation of the Therapeutic Effect of TPP‐SAT in an Animal Model of Periodontitis

**Figure 5:**
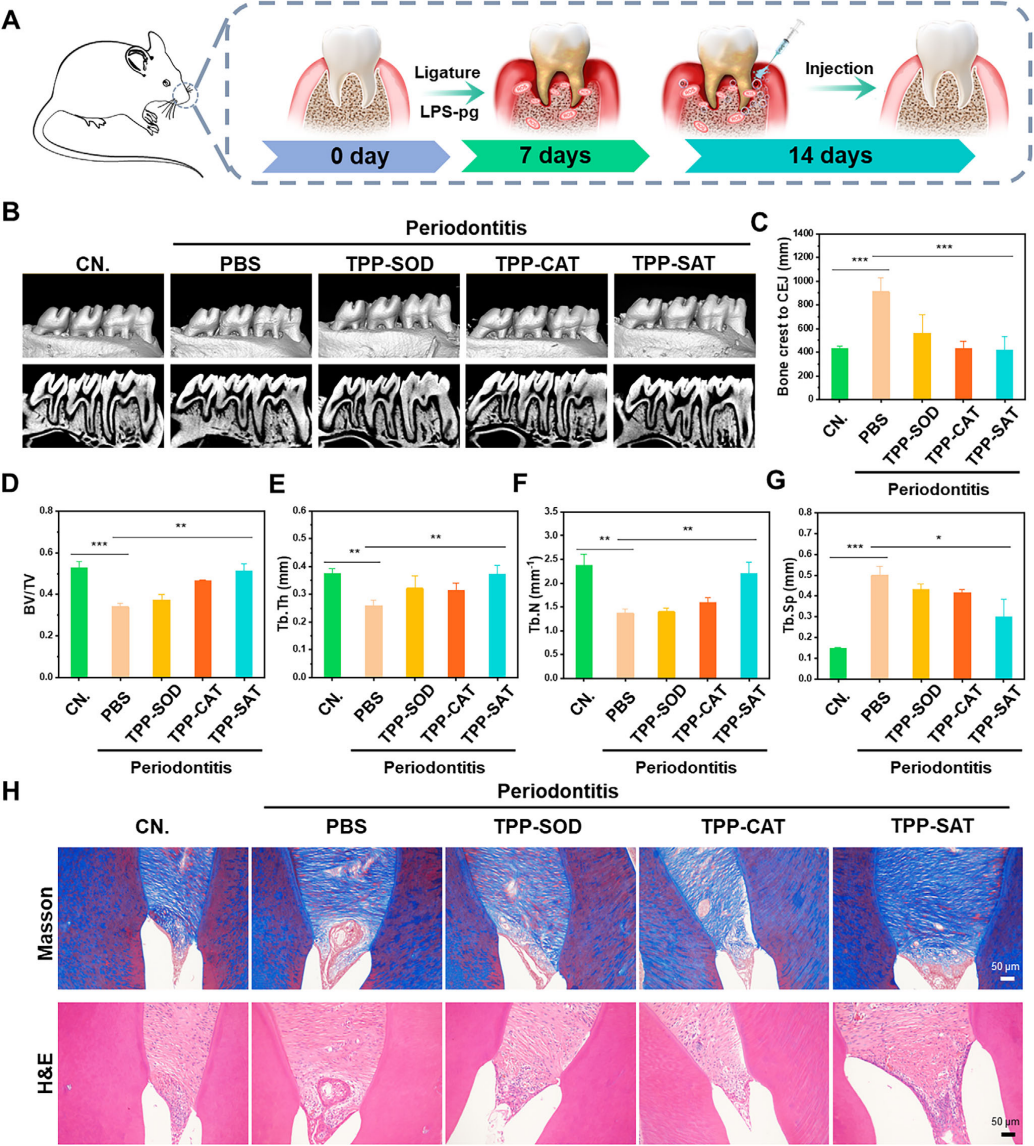
Validation of the therapeutic effect of TPP‐SAT in an animal model of periodontitis. A) Establishment of a periodontitis model and schematic representation of treatment strategies. B) Micro‐CT reconstructed 3D images and buccopalatal cross‐sections of maxillary molars under various treatment conditions. C) Quantitative evaluation of the distance between ABC and CEJ. Microarchitectural parameters of alveolar bone were quantified, including D) BV/TV, E) Tb.Th, F) Tb.N, and G) Tb.Sp. H) Masson's trichrome and H&E staining of rat periodontal tissues from different drug treatment groups (Scale Bar: 50 µm). Data are presented as the mean ± standard deviation (SD). Statistical significance was calculated by t‐test for comparison between two groups. ns: no significant, *p < 0.05, **p < 0.01, ***p < 0.001.

The evidence for the therapeutic potential of TPP‐SAT for periodontitis was based on the material level as well as the cellular level. Subsequently, TPP‐SAT was used for the treatment of animal models of periodontitis. The animal model was established and the treatment protocol is shown in **Figure** **5**A. Micro‐computed tomography (Micro‐CT) analysis were used to assess the treatment effect. And the vertical distance between the alveolar bone crest (ABC) and the cementoenamel junction (CEJ) was considered as an indicator of treatment by 3D reconstruction. As shown in Figure 5B,C, the PBS group had the longest distance between the ABS and CEJ compared to the control group and the other treatment groups, implying the most severe alveolar bone loss. In contrast, the distance between ABS and CEJ in the TPP‐SAT group was significantly decreased compared to the PBS group and also significantly decreased compared to the single treatment group. This indicates that TPP‐SAT exhibits a significant therapeutic effect on the symptoms of periodontitis. Moreover, compared to the PBS group, the TPP‐SAT treatment group exhibited increased BV/TV, Tb.Th, and Tb.N, along with decreased Tb.Sp, indicating that TPP‐SAT promotes periodontal tissue repair and facilitates recovery from periodontitis (Figure 5D–G). The above experimental results proved the successful establishment of periodontitis model and the significant effect of TPP‐SAT on the treatment of periodontitis.

Futhermore, periodontal tissue sections from different treatment groups were subjected to Masson's trichrome and hematoxylin&eosin (H&E) staining. Histological analysis revealed that PBS‐treated samples exhibited significant collagen depletion and prominent inflammatory cell infiltration. In contrast, TPP‐SAT‐treated samples showed markedly enhanced collagen deposition and a substantial reduction in inflammatory cells. (Figure 5H).

**Figure 6:**
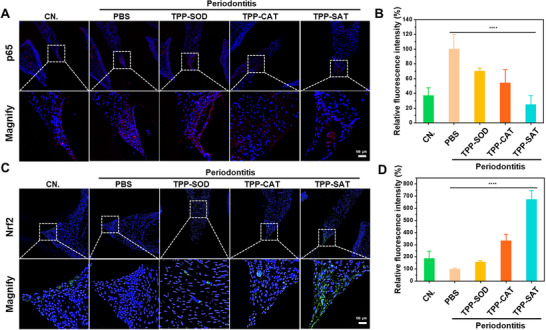
Immunofluorescence staining and histological staining of periodontal tissue sections from different dosing groups. A) Immunofluorescence staining of P65 protein in periodontal tissue (Scale Bar: 100 µm). B) Quantitative fluorescence analysis of p65 expression across treatment groups in Figure 6A. C) Immunofluorescence staining of Nrf2 protein in periodontal tissue (Scale Bar: 100 µm). D) Quantitative fluorescence analysis of Nrf2 expression across treatment groups in Figure 6C. Data are presented as the mean ± standard deviation (SD). Statistical significance was calculated by t‐test for comparison between two groups. ns: no significant, ****p < 0.0001.

Subsequently, the therapeutic efficacy of TPP‐SAT for periodontitis was further validated by immunological staining as well as histological staining of periodontal tissues. To further explore the antioxidant properties of TPP‐SAT in vivo, periodontal tissue sections from different dosing groups were used for P65 as well as Nrf2 immunofluorescence staining. As shown in **Figure** **6A‐D**, there was a significant decrease in the expression level of P65 and a significant increase in the expression of the antioxidant protein Nrf2. This demonstrates that TPP‐SAT has excellent antioxidant properties in vivo. Finally, the biosafety of TPP‐SAT in vivo was verified by H&E staining, and it was found that there were no obvious toxic side effects on any of the major organs, which provided strong support for the clinical translation of TPP‐SAT (Figure S7, Supporting Information).

## 3 Conclusions

In this study, we developed a mitochondria‐targeted natural enzyme cascade nanogel (TPP‐SAT) with efficient ROS‐scavenging capability. TPP‐SAT selectively targets mitochondrial ROS and converts harmful species such as H_₂_O_₂_ and ·O_₂_^⁻^ into therapeutic O_₂_ through a cascade enzymatic reaction. This process not only alleviates local hypoxia and reduces oxidative stress but also modulates immune responses by rebalancing M1/M2 macrophage polarization. Following periodontal injection, TPP‐SAT regulates the expression of cellular p65 and the Nrf2/NF‐κB signaling pathways, enhances the antioxidant defense system, and significantly promotes the repair of inflamed periodontal tissues. In summary, this study presents a biocompatible and effective natural enzyme‐based therapeutic strategy with strong clinical translational potential for the treatment of periodontitis.

## 4 Experimental Section

### Materials

Superoxide dismutase (SOD), catalase (CAT), Horseradish peroxidase (HRP), 2‐(Dimethylamino)ethyl methacrylate (2‐DM), bisacrylamide, acrylamide, allylphosphine bromide (TPP), ammonium persulfate (APS) and N,N,N“,N”‐tetramethylethylenediamine (TEMED) were obtained from Sigma–Aldrich. BCA (bicinchoninic acid) protein assay was purchased from Thermo. Antifade mounting medium with DAPI (4′,6‐diamidino‐2‐phenylindole), Annexin V‐FITC/PI Apoptosis Detection Kit and DCFH‐DA (dichlorodihydrofluorescein diacetate) were obtained from Beyotime Biotechnology. iNOS antibody (CL647‐18985) and Arg‐1 antibody (CL594‐66129) were obtained from Proteintech. Phosphate buffered saline (PBS) and JC‐1 (5′, 6, 6′‐tetrachloro‐1, 1′, 3, 3′‐tetraethylbenzimidazolylcarbocyanine iodide) was supplied by Solarbio Technology Co., Ltd. [Ru (dpp)_3_]Cl_2_ (Luminescent oxygen sensor) was purchased from Shanghai Maokang Biotechnology Co., Ltd. NF‐κB p65 (D14E12) XP Rabbit mAb #8242 and NRF2 (D1Z9C) XP Rabbit mAb #12 721 was purchased from Cell Signaling Technology. Anti‐Mouse CD86 Rabbit Recombinant Antibody and Anti‐Mouse CD206 Rabbit Recombinant Antibody was purchased from Proteintech Group, Inc. Mouse IL‐10 Uncoated ELISA Kit (88‐7105‐88), Mouse TNF alpha Uncoated ELISA Kit (88‐7324‐88) and Mouse IL‐10 Uncoated ELISA Kit (88‐7105‐88) were purchased from Thermo Fisher Scientific Inc.

### Synthesis of TPP‐SAT Nanogel

The synthesis of TPP‐SAT was carried out as follows: 2‐DM was added to the SOD and CAT solution (SOD:CAT = 1:1, w/w) at a molar ratio of 10 000:1 to the CAT enzyme and stirred at room temperature for 6 h. Allylphosphine bromide, acrylamide and bisacrylamide were then added to the mixture at molar ratios of 100:1, 10 000:1 and 5000:1 to CAT, respectively, and stirred for 1 h under N_2_ protection. Then, ammonium persulfate (APS:CAT = 550:1, mol:mol) was dissolved in 1 mL of deionized water, and N,N,N“,N”‐tetramethylethylenediamine (TEMED:APS, 1.8:1, w/w) was added to initiate free radical polymerization on the surface of the acylated protein, and the reaction was continued for 1 h under N_2_ protection. Finally, Finally, the final TPP‐SAT nanogel was collected by dialysis and freeze‐drying.

### Free Radical Scavenging by TPP‐SAT In Vitro

To investigate the efficiency of TPP‐SAT in scavenging H_2_O_2_ in vitro, we verified the scavenging of free radicals by TMB colorimetric reaction. Briefly, 5 µL (1 mg mL^−1^) of TPP‐SAT was added to 200 mM H_2_O_2_ solution and reacted at 37 °C for 10 min. Subsequently, 2 µL of HRP (0.06 mg mL^−1^) and 100 µL of color development solution (250 µL of TMB in 1 mL of PBS) were added and mixed for 15 min and the absorption spectra were measured at the wavelengths of 550–730 nm. In addition, in order to verify the ability of TPP‐SAT to clear superoxide radicals, its activity to clear superoxide radicals was verified according to the experimental procedure of the total SOD activity assay kit (Beyotime, S0101S).

### Characterization of TPP‐SAT Nanogel

The morphology of TPP‐SAT nanogel was characterized by TEM (G2 Spirit Biotwin, FEI) Hydrated particle size was characterized by Zetasizer Nano ZS90. In addition, the structure of TPP‐SAT, SAT and TPP was characterized by FTIR (Microlet MICOET iS10).

### Cell Culture

The RAW 264.7 cells, L929 cells and PDLSCs cells were cultured in DMEM with 10% FBS and 1% penicillin and streptomycin in a humidified incubator containing 5% CO_2_ and maintain at 37 °C. In addition, the HUVEC cells were cultured in endothelial cell complete medium in a humidified incubator containing 5% CO_2_ and maintain at 37 °C.

### Cytotoxicity Evaluation

To validate the potential application of TPP‐SAT, MTT assay, live‐dead cell staining, and apoptosis assay were used to evaluate the biosafety of TPP‐SAT. For MTT assay, 1×10^4^ RAW 264.7, PDLSCs and HUVEC cells were cultured overnight in inoculated 96‐well cell culture dishes, respectively. And 0–40 µg mL^−1^ TPP‐SAT were dissolved in medium and added into the culture dishes, after 18 h of incubation, MTT experiments were subsequently performed. For live‐dead cell staining assay, 1×10^6^ RAW 264.7 cells were cultured overnight in inoculated 6‐well cell culture dishes, and 10 µg mL^−1^ TPP‐SOD, TPP‐CAT and TPP‐SAT were dissolved in medium and added into the culture dishes, after 18 h of incubation, staining of cells by live‐dead cell staining kits and the cells state were observed in inverted fluorescence microscope (Leica DMi8). For apoptosis assay, the experimental procedures were similar to the live‐dead staining experiments, with the difference that the cells were stained by the apoptosis detection kit and detected in a flow cytometer (Beckman CytoFLEX LX).

### Mitochondria Targeted of SAT and TPP‐SAT

To verify that TPP‐SAT has the ability to target cellular mitochondria and scavenge free radicals. 5×10^4^ RAW 264.7 cells were first inoculated in 48‐well plates pre‐laid with cell crawlers and cultured overnight. Subsequently, 10 µg mL^−1^ of SAT and TPP‐SAT were added and co‐incubated for 10 h. Subsequently, the plates were sealed with an anti‐fluorescence quenching sealer containing DAPI, and the mitochondrial targeting abilities of SAT and TPP‐SAT were observed in a confocal Laser Microscope (CLSM) (Zeiss LSM5).

### Intracellular Reactive Oxygen Species Detection

Intracellular reactive oxygen species (ROS) levels were characterized by the ROS fluorescent probe DCFH‐DA. The increase of fluorescence intensity indicated the increase of ROS level. In brief, 5×10^4^ RAW 264.7 cells were first inoculated in 48‐well plates pre‐laid with cell crawlers and cultured overnight with 100 µM H_2_O_2_. And then 10 µg mL^−1^ TPP‐SOD, TPP‐CAT and TPP‐SAT were added into medium. After 10 h of incubation, the culture medium was replaced with DCFH‐DA stock solution (10 nM) and incubated for 30 min. Subsequently, the plates were sealed with an anti‐fluorescence quenching sealer containing DAPI, and the ROS were observed in a CLSM. The experimental procedure for ROS detection by flow cytometry is similar to that described above, with the difference that it does not involve staining of the nucleus.

### Detection of Intracellular Oxygen Production

The concentration of intracellular oxygen is measured primarily by the oxygen fluorescent probe. A decrease in fluorescence intensity indicates an increase in oxygen concentration. In brief, 5×10^4^ RAW 264.7 cells were first inoculated in 48‐well plates pre‐laid with cell crawlers and cultured overnight with 100 µM H_2_O_2_. And then 10 µg mL^−1^ TPP‐SOD, TPP‐CAT and TPP‐SAT were added into medium. After 10 h of incubation, the culture medium was replaced with [Ru(dpp)^3^]Cl_2_ stock solution (10 µg mL^−1^) and incubated for 30 min. Subsequently, the plates were sealed with an anti‐fluorescence quenching sealer containing DAPI, and the fluorescence were observed in a CLSM.

### Intracellular Mitochondrial Membrane Potential Detection

Changes in intracellular mitochondrial membrane potential (MMP) were mainly reflected by the fluorescence shift of the fluorescent probe JC‐1. Mitochondrial activity was assessed by green fluorescence (JC‐1 monomer) and red fluorescence (JC‐1 aggregates) using 535 and 595 nm emission filters, respectively. In brief, 5×10^4^ RAW 264.7 cells were first inoculated in 48‐well plates pre‐laid with cell crawlers and cultured overnight with 100 µM H_2_O_2_. And then 10 µg mL^−1^ TPP‐SOD, TPP‐CAT and TPP‐SAT were added into medium. After 10 h of incubation, JC‐1 (5 µg mL^−1^) were added into medium and incubated 20 min. Subsequently, the plates were sealed with an anti‐fluorescence quenching sealer containing DAPI, and the fluorescence were observed in a CLSM.

### Angiogenesis Assay in HUVEC Cells

The ability of TPP‐SAT to promote angiogenesis was verified by HUVEC cells. In brief, 100 µL of matrix gel was added to a 24‐well plate on ice bath conditions and incubated at 37 °C for 15 min. And 1×10^5^ HUVEC cells were inoculated in the medium with 100 µM H_2_O_2_. Subsequently, 10 µg mL^−1^ TPP‐SOD, TPP‐CAT and TPP‐SAT were added into medium. After 10 h of incubation, The HUVEC cells were stained by Annexin V‐FITC and observed in an inverted fluorescence microscope.

### Immunofluorescent Staining Experiment

The immunofluorescence experiments involved in the experiment were carried out according to the following experimental procedure. Briefly, 5×10^4^ RAW 264.7 cells were spread into 48‐well plates pre‐lined with cell crawls and cultured overnight with 100 µM H_2_O_2_. The cells were then incubated with 10 µg mL^−1^ of TPP‐SOD, TPP‐CAT, TPP‐SAT for 10 h. The cells were fixed, washed and incubated with primary antibody overnight, followed by secondary antibody incubation for 2 h. DAPI staining and blocking were performed in CLSM for observation.

### Flow Cytometric Analysis of Macrophage Phenotypes

1×10^6^ RAW 264.7 cells were lined up in 6‐well plates and incubated with 100 ng mL^−1^ of LPS for 24 h. The original culture medium was aspirated and replaced with serum‐free medium, and 10 µg mL^−1^ of TPP‐SOD, TPP‐CAT, and TPP‐SAT were added to incubate the cells for 10 h. Afterwards, the cells were collected, the membrane was broken and stained for CD11b (macrophage marker), CD206 (M2 macrophage marker) and CD86 (M1 macrophage marker) proteins respectively. Finally, the cells were analyzed by flow cytometry.

### Enzyme‐Linked Immunosorbent Assay Experiment

Detection of cytokines such as IL‐6, IL‐10, TNF‐α and TGF‐β is performed according to the enzyme‐linked immunosorbent assay (ELISA) kit procedure.

### Establishment of Rat Periodontitis Model

Rats of the WISTAR (6‐7 weeks) strain were ordered from the Xiamen University Laboratory Animal Center. The experiments were approved by the Animal Management and Ethics Committee of Xiamen University (Animal experiment ethical review number: XMULAC20220077). To form rat periodontitis model, the rats were divided into blank control group, periodontitis group, TPP‐SOD group, TPP‐CAT group and TPP‐SAT group. Intraperitoneal anesthesia with 0.3% sodium pentobarbital was used at a dose of 50 mg kg^−1^ body weight. An orthodontic ligature wire of 0.4 mm in diameter was fixed around the left second molar in the maxilla, and periodontitis was induced by injection of LPS‐PG (100 ng mL^−1^) at the second molar site. After one week, the ligature wire was removed from the neck of the molar and considered as periodontitis model establishment. After two weeks of treatment, they were executed and the next experiment was carried out.

### Microcomputed Tomography (CT) Scanner and Histologic Evaluation

To assess bone loss, hard tissues including the maxilla and teeth were imaged using a high‐resolution micro‐CT imaging system (SkyScan 1272). The vertical distance from the alveolar bone crest (ABC) of the maxillary second molar to the cementoenamel junction (CEJ) was measured by 3D reconstructed CT images as an indicator of bone loss. Histologic analysis of the maxillary tissues was performed using hematoxylin and eosin (H&E) staining to assess inflammation and Masson trichrome staining to assess tissue repair.

### Statistical Analysis

Values are shown as mean ± standard deviation, a GraphPad Prism 8.0.2 software and ImageJ were applied for statistical analysis. Multiple t tests were used for statistical analysis to compare statistical significance. ^*^p < 0.05, ^**^p < 0.01 and ^***^p < 0.001, ^****^p < 0.0001 were respectively recognized as statistically significant, highly significant, and very significant.

## Conflict of Interest

The authors declare no conflict of interest.

## Supporting information



Supporting Information

## Data Availability

The data that support the findings of this study are available from the corresponding author upon reasonable request.
