# Supplementary material for: Mitochondria‐Targeted ROS Scavenging Natural Enzyme Cascade Nanogels for Periodontitis Treatment via Hypoxia Alleviation and Immunomodulation
Source: Adv Sci (Weinh). 2025 May 23;12(29):e07481. doi: 10.1002/advs.202507481 (PMC12362747; doi:10.1002/advs.202507481)
Supplement: Supplementary file 1 — Supporting Information [file ADVS-12-e07481-s001.docx]

**Supporting Information**

for

**Mitochondria-targeted ROS Scavenging Natural Enzyme Cascade Nanogels for Periodontitis Treatment via Hypoxia Alleviation and Immunomodulation**

Yanfen Zheng^a,c,1^, Liuzhou Mao^b,1^, Qi Wang^b^, Haohua Hu^b^, Bahriman Xarpidin^b^, Zheng Luo^b,^*, Yun-Long Wu^b,^*

^a^ Xiamen Key Laboratory of Stomatological Disease Diagnosis and Treatment, Stomatological Hospital of Xiamen Medical College, Xiamen 361008, China

^b^ Fujian Provincial Key Laboratory of Innovative Drug Target Research and State Key Laboratory of Cellular Stress Biology, School of Pharmaceutical Sciences, Xiamen University, Xiamen 361102, China

^c^ Engineering Research Center of Fujian University for Stomatological Biomaterials

E-mail address: wuyl@xmu.edu.cn (Y.-L. Wu), [zhluo1994@163.com](mailto:zhluo1994@163.com) (Z. Luo)

^1^ The authors contributed equally to this work.


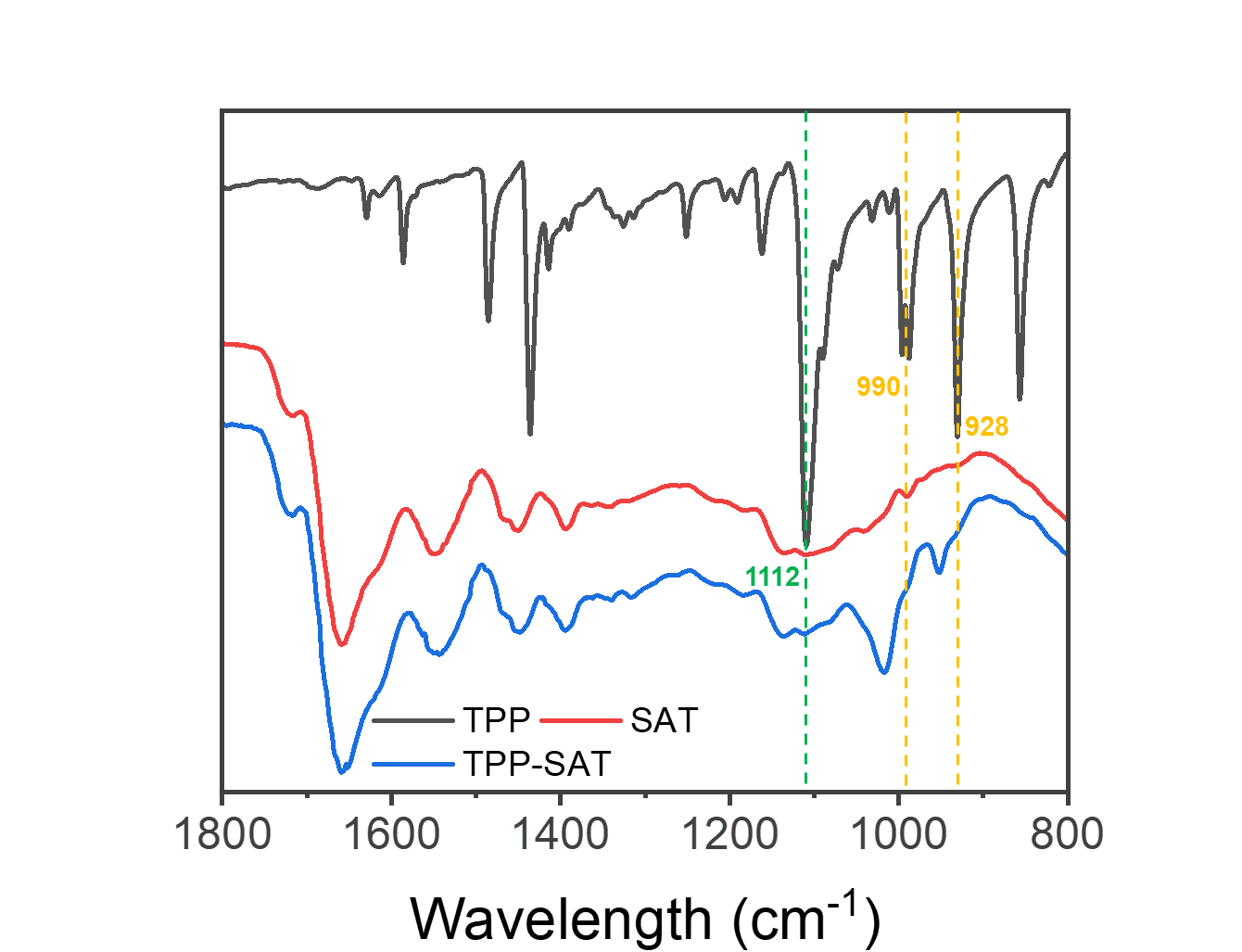


**Figure S1:** The Fourier-transform infrared (FTIR) spectra of TPP, SAT and their composite (TPP-SAT).

**
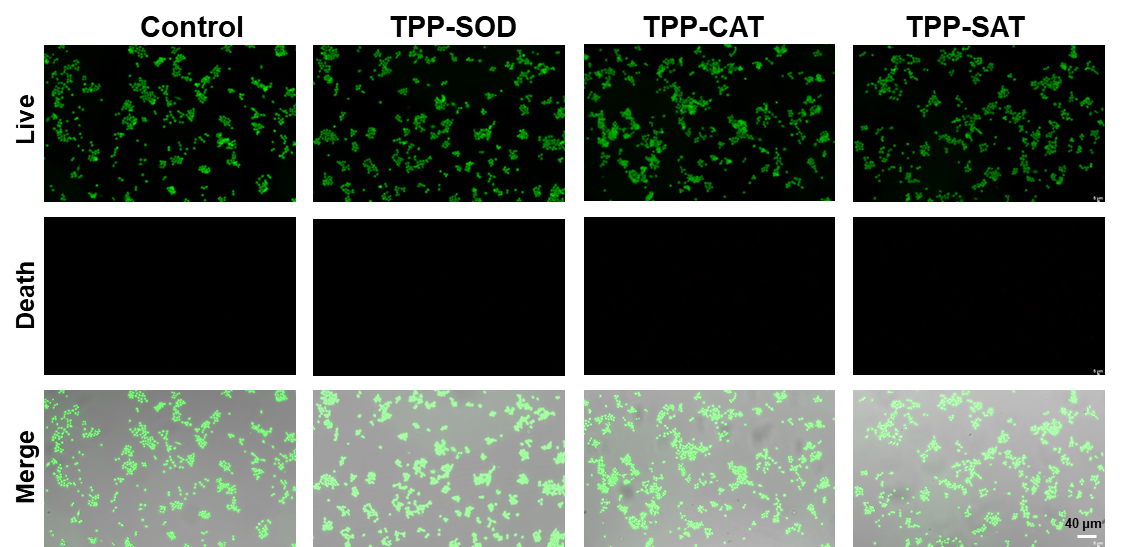
**

**Figure S2:** Fluorescence micrographs of live/dead staining in RAW 264.7 cells treated with different materials (Scale bar: 40 μm).​


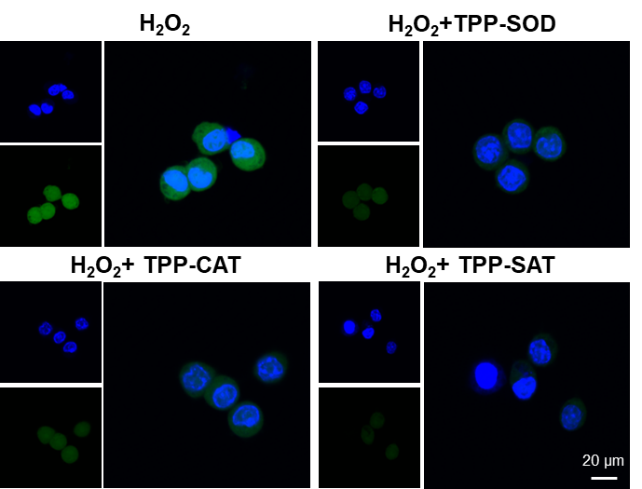


**Figure S3:** Fluorescence micrographs of ROS staining in RAW 264.7 cells treated with different materials (Scale bar: 20 μm).

**
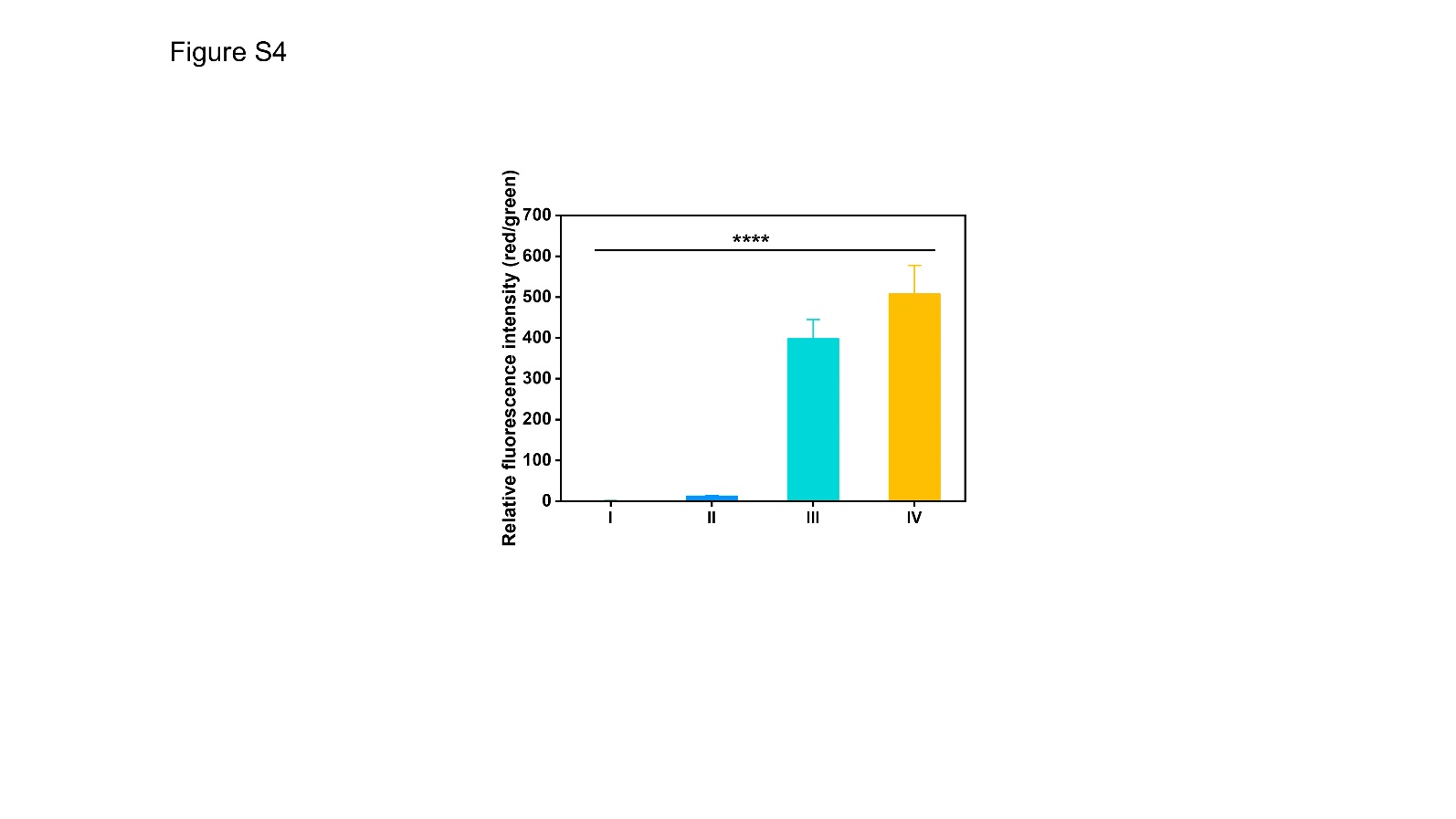
**

**Figure S4:** Quantitative detection of mitochondrial membrane potential in RAW 264.7 cells treated with different materials. (Ⅰ. H_2_O_2_; Ⅱ. H_2_O_2_+TPP-SOD; Ⅲ. H_2_O_2_+TPP-CAT; Ⅳ. H_2_O_2_+TPP-SAT.)

**
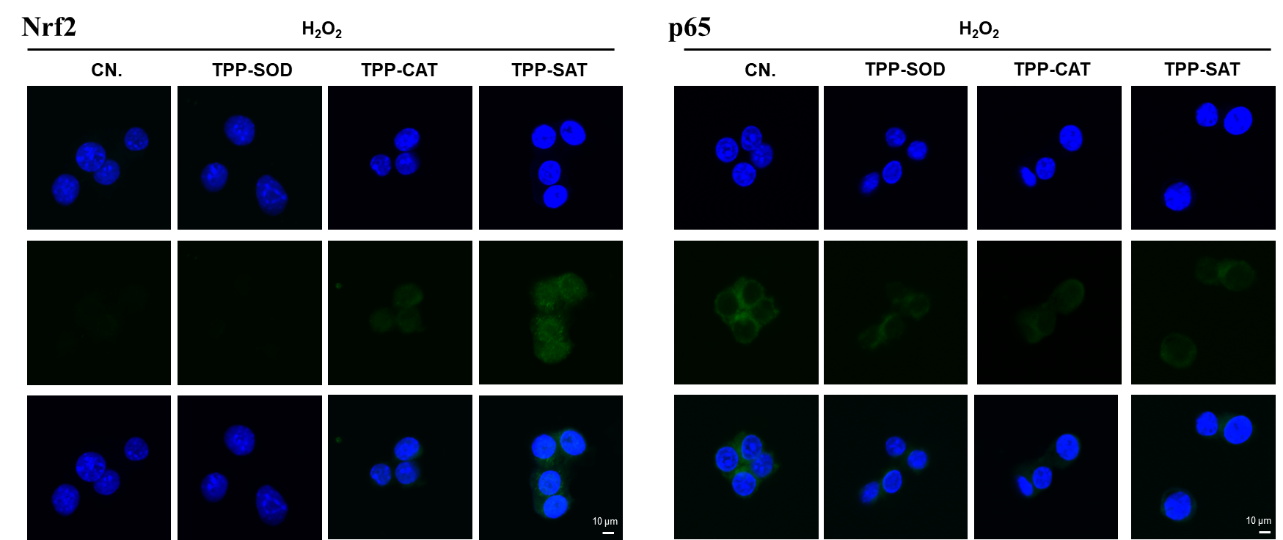
**

**Figure S5:** Fluorescence micrographs showing p65 and Nrf2 expression in RAW 264.7 cells treated with different materials (Scale bar: 10 μm).


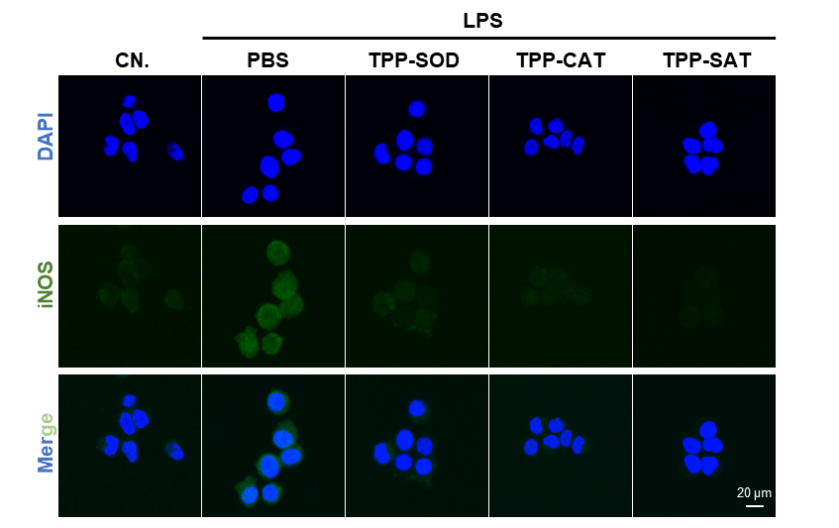


**Figure S6:** Fluorescence micrographs showing iNOS expression in RAW 264.7 cells treated with different materials (Scale bar: 20 μm).​

**
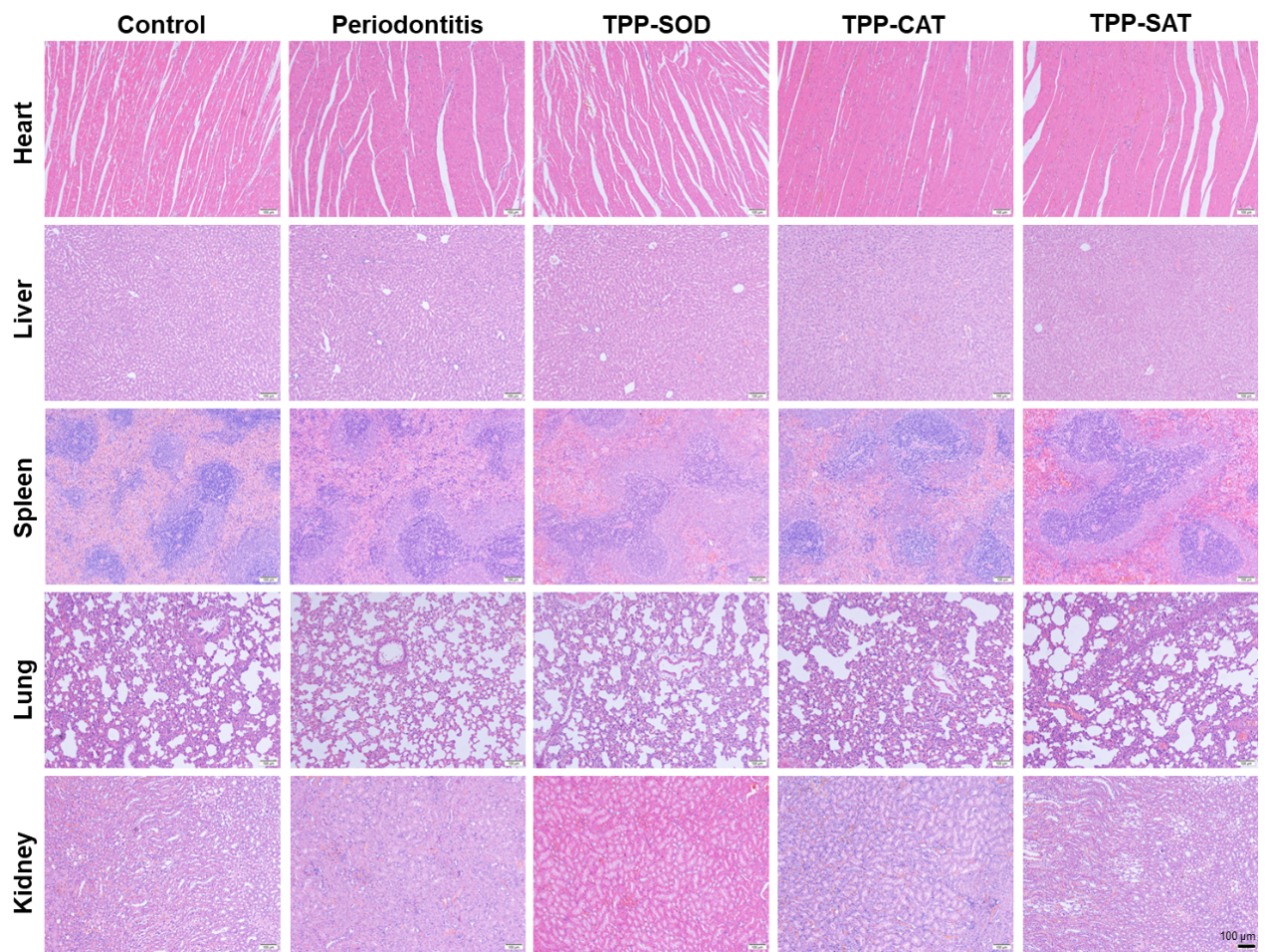
**

**Figure S7:** H&E staining of major organs in rats treated with different drug administrations (Scale bar: 100 μm).
